# Sex differences during a cold-stress test in normobaric and hypobaric hypoxia: A randomized controlled crossover study

**DOI:** 10.3389/fphys.2022.998665

**Published:** 2022-09-23

**Authors:** Erich Hohenauer, Wolfgang Taube, Livia Freitag, Ron Clijsen

**Affiliations:** ^1^ Rehabilitation and Exercise Science Laboratory (RES lab), Department of Business Economics, Health and Social Care, University of Applied Sciences and Arts of Southern Switzerland, Landquart, Switzerland; ^2^ Department of Physiotherapy, International University of Applied Sciences THIM, Landquart, Switzerland; ^3^ Department of Neurosciences and Movement Science, University of Fribourg, Fribourg, Switzerland; ^4^ Department of Movement and Sport Sciences, Vrije Universiteit Brussel, Brussels, Belgium; ^5^ Department of Health, Bern University of Applied Sciences, Berne, Switzerland

**Keywords:** hypoxia, cold, physiology, cold and hypoxia, sex difference

## Abstract

Cold and hypoxia are two stressors that are frequently combined and investigated in the scientific literature. Despite the growing literature regarding normobaric hypoxia (NH) and hypobaric hypoxia (HH), responses between females and males are less often evaluated. Therefore, this study aims to investigate the physiological sex differences following a cold-stress test under normoxia, normobaric- and hypobaric hypoxia. A total of *n* = 10 females (24.8 ± 5.1 years) and *n* = 10 males (30.3 ± 6.3 years) from a university population volunteered for this study. The cold-stress test (CST) of the right hand (15°C for 2 min) was performed using a randomised crossover design in normobaric normoxia, NH and HH. The change (∆) from baseline to post-CST up to 15 min was analysed for cutaneous vascular conductance (CVC) and the hands’ skin temperature, whilst the mean values across time (post-CST up to 15 min) were assessed for peripheral oxygen saturation (SpO_2_), thermal sensation- and comfort. Pressure pain threshold (PPT) was assessed after the post-CST 15 min period. The hands’ skin temperature drop was higher (*p* = 0.01) in the female group (∆3.3 ± 1.5°C) compared to the male group (∆1.9 ± 0.9°C) only in NH. Females (−0.9 ± 0.5) rated this temperature drop in NH to feel significantly colder (*p* = 0.02) compared to the males (−0.2 ± 0.7). No differences were observed between sexes in NN, NH, and HH for ∆CVC, SpO_2_, thermal comfort and PPT. In conclusion, females and males show similar reactions after a CST under normoxia and hypoxia. Sex differences were observed in the local skin temperature response and thermal sensation only in NH.

## 1 Introduction

Early studies from the 1950s have already demonstrated, that cold stimulus provokes action potentials in peripheral nerve endings (Hensel and Zotterman, 1951). Later studies then demonstrated, that cold induces a Ca^2+^ influx, suggesting direct opening of Ca^2+^-permeable ion channels by this thermal stimulus (Suto and Gotoh, 1999; Reid and Flonta, 2002; Reid, 2005). The physiological response to local cooling is undoubtedly a vasoconstriction, which is from a functional perspective part of a thermoregulatory homeostatic response to reduce heat loss (Cankar and Finderle, 2003). Although several mechanisms are involved to minimize heat loss via control of skin blood flow, an intact sympathetic system is necessary for the initial reflex response (Ekenvall et al., 1988; Johnson et al., 2005). In case of the cold-activated vasoconstrictive system, the transmitters appear to be norepinephrine and other co-transmitters (Morris, 1999; Stephens et al., 2001; Stephens et al., 2004). Inhibition of NO system, postsynaptic upregulation of alpha_2c_-receptors and cold-sensitive afferents seem to be important elements to induce vasoconstriction (Johnson and Kellogg, 2010). Although, peripheral vasoconstriction is a powerful mechanism to reduce heat loss, it can lead to a severe temperature drop in the extremities as skin blood flow reduces (Daanen, 2003). After initial vasoconstriction, cold-induced vasodilation occurs as a physiological reaction to local cold exposure, followed by another vasoconstriction (Daanen, 2003). Cyclic opening of arterio-venous anastomoses, located in the fingers and toes, allows the maintenance of blood flow and thus minimizing cold-induced injuries (Cheung, 2015). To evoke this so called cold-induced vasodilation, cold-water is most commonly used due to its high thermal conductivity compared to e.g., air (Smith and Hanna, 1975). The sympathetic activation and increased peripheral resistance will also alter heart-rate and blood pressure (Cui et al., 2002). The discharge of norepinephrine triggers the cardiovascular system, leading to arteriolar constriction, increased heart-rate and increased cardiac contractility (Silverthorn and Michael, 2013). These combined responses increase blood-pressure and are known as the pressor response (Velasco et al., 1997). Beside cardiovascular responses, cold applications are also known to have an influence on sensory perception, especially pain perception. It is well known, that tissue temperature reductions impact sensory and motor nerve conduction velocity (Herrera et al., 2011). Cold water-immersions, due to its high thermal conductivity, can significantly reduce nerve-conduction velocity to induce a hypoalgesic effect (Herrera et al., 2010).

Cold exposure can naturally occur in combination with hypoxia during exposure to terrestrial altitude. Hypoxia research has numerous applications, investigating pathogenesis but also for developing medical treatment strategies (Grocott et al., 2007; Saxer et al., 2019; Schneider et al., 2022). The potential physiological difference between normobaric hypoxia (NH) and hypobaric hypoxia (HH) is currently a topic of much debate (Millet and Debevec, 2020; Richalet, 2020). It would be expedient to assume that NH is a surrogate for HH (Conkin, 2016). However, conditions with a different fraction of inspired oxygen (F_i_O_2_) and barometric pressure having the same partial pressure of oxygen (P_i_O_2_) aren’t interchangeable (Conkin, 2016). For example, ventilatory responses, fluid balance, acute mountain sickness, nitric oxide metabolism and physical performance differed between these conditions and suggest that HH is a more severe environment compared to NH (Millet et al., 2012). In accordance to these results, HH has been demonstrated to induce a more pronounced sympathetic effect compared to NH, probably due to an increased ventilatory stimulus and larger desaturation (Aebi et al., 2020a). The increased ventilatory response in HH compared to NH was also observed by the same research group in another study (Aebi et al., 2020b). Contradictory, no differences between NH and HH have been observed regarding baroreflex sensitivity and the authors concluded, that these conditions might be used interchangeably to assess this outcome (Bourdillon et al., 2017). Indeed, other physiological outcomes like ventilatory responses, cardiovascular variables and arterial partial pressure have also been demonstrated not to differ between NH and HH (Naughton et al., 1995; Savourey et al., 2007; Faiss et al., 2013). In a review study, it was highlighted that the presence of confounding factors such as time spent in hypoxia, temperature, humidity and small sample sizes might limit profound conclusions (Coppel et al., 2015).

Despite these findings, evidence seems to be lacking with respect to hypoxia on thermal perception in humans (Golja et al., 2004). Additionally, the manner in which men and women respond differently to physiological stressors are less well investigated (Miller et al., 2019). The reduction in barometric pressure and as a result the reduction in F_i_O_2_ lead to a decline in arterial oxygen saturation (SpO_2_) (Rowell et al., 1989). Whilst observing comparable O_2_ desaturation declines in both sexes, males tend to have a higher sympathetic activation compared to females under hypoxia (Botek et al., 2018). Some authors have associated symptoms of acute mountain sickness with sympathetic dominance in autonomic cardiac control (Sutherland et al., 2017). Studies demonstrated that young females have a different vasoconstrictive response compare to men in hypoxia, possible due to female sex hormones (Patel et al., 2014). From this perspective, it’s thinkable, that cardiovascular reactions to cold exposure also might differ between sexes. The physiological reactions to external stressors are dependent on an intact nervous system and hypoxia has been demonstrated to affect various physiological systems in the human body (Seech et al., 2020; Blacker and McHail, 2022). Accordingly, it is thinkable that hypoxia might also affect thermoregulatory reactions and thermal sensitivity. Despite progression in physiological research, male-only studies are predominantly present in this area, compared to female-only studies (4:1) or don’t take the sex difference into account when interpreting the results (Beery and Zucker, 2011; Ansdell et al., 2020). As a result, future research is needed to investigate sex related differences (Lamotte et al., 2021). Therefore, the aim of this study is to evaluate the physiological sex differences following a cold-stress test under normoxia, normobaric- and hypobaric hypoxia. Thus, we want to contribute to highlighting the importance of research diversity in this area.

## 2 Materials and methods

### 2.1 Participants

A total of *n* = 10 females (24.8 ± 5.1 years, 62.8 ± 6.4 kg, 168.6 ± 6.2 cm) and *n* = 10 males (30.3 ± 6.3 yrs, 84.5 ± 9.8 kg, 182.1 ± 5.4 cm) were recruited from a university population in Switzerland. The participants were healthy, non-smokers, recreationally trained, and free of any known medical disorders. Participants were excluded in case of pregnancy, exposure to altitude >1,000 m (including commercial flights) for at least 1 month before the start of the experiments, or if they have ever experienced any altitude-related negative effects (e.g., acute mountain sickness).

Participants were instructed to refrain from alcohol and other substances that might affect the cardiorespiratory system, 72 h before the start of the experiment. The participants were instructed to maintain their normal food and water intake and to replicate these habits for each subsequent trial. This study was approved by the Ethical Committee of Zurich (project-ID:2019-00504) in accordance with the Declaration of Helsinki (ICH-GCP), registered (ClinicalTrial.gov Identifier: NCT04075565) and all participants provided their written informed consent before participation.

### 2.2 Experimental design

This study employed a randomized controlled crossover design and was completed within 3 months to ensure that the females were at the same menstrual phase in each trial. During NN, the participants performed the experiment under laboratory conditions, at ∼550 m. During NH, participants were exposed to a F_i_O_2_ of 0.1440 (equivalent to ∼3,000 m) in the same laboratory, 15 min before the experimental baseline measurements were conducted. The NH environment was created using a hypoxic generator (Cloud 9, sporting edge UK LTD Basingstoke, United Kingdom). For the hypobaric hypoxia (HH) environment, participants conducted the experiments in terrestrial altitude at a height of ∼3,000 m at a mountain hut in the Swiss Alps. The transportation of the participants (car and cable car) to the mountain hut took about 135 min.

The participants were blinded to the NN and NH conditions, which was confirmed by the exit questionnaire. During NN, the participants wore the mask system from the altitude generator but the tube was not connected to the running hypoxic generator. The environmental calculations for the females and males are shown in Table 1.

**TABLE 1:** Environmental conditions. *N* = 20, one-way ANOVA.

	P_i_O_2_ (mmHg)	F_i_O_2_ (fraction)	P_B_ (mmHg)	PH_2_O (mmHg)	RT (°C)	*p*-value
NNm	146.5 ± 1.3	0.2093	720.6 ± 6.0	20.9 ± 0.8	22.8 ± 0.7	All >0.05
NNf	145.5 ± 1.6	0.2093	715.4 ± 7.6	20.4 ± 0.7	22.5 ± 0.5	
NHm	100.9 ± 0.6	0.1440	721.1 ± 4.4	20.5 ± 0.5	22.6 ± 0.4	All >0.05
NHf	100.6 ± 0.8	0.1440	719.1 ± 6.0	20.1 ± 0.8	22.2 ± 0.7	
HHm	105.7 ± 0.4	0.2093	522.6 ± 1.3	17.4 ± 2.0	20.0 ± 2.1	All >0.05
HHf	105.4 ± 0.4	0.2093	522.2 ± 0.4	18.6 ± 1.9	21.0 ± 1.6	

Abbreviations: NN, normobaric normoxia; NH, normobaric hypoxia; HH, hypobaric hypoxia; m, males; f, females; P_i_O_2_, inspired oxygen tension; F_i_O_2_, fraction of inspired oxygen; P_B_, barometric pressure; PH_2_O, water vapour pressure; RT, room temperature, values are means ± SD.

### 2.3 Study overview

Before their first visit, participants were familiarized with the procedures, which were carried out in a seated position throughout the experiment. Before each trial, participants were instrumented and rested for the duration of 15 min before the data collection started. In NH, the participants were already breathing the hypoxic air during this time period. Then, SpO_2_, local skin temperature of the hand, microcirculation of the hand, blood pressure, thermal sensation, and thermal comfort were assessed (all described later).

**FIGURE 1:**
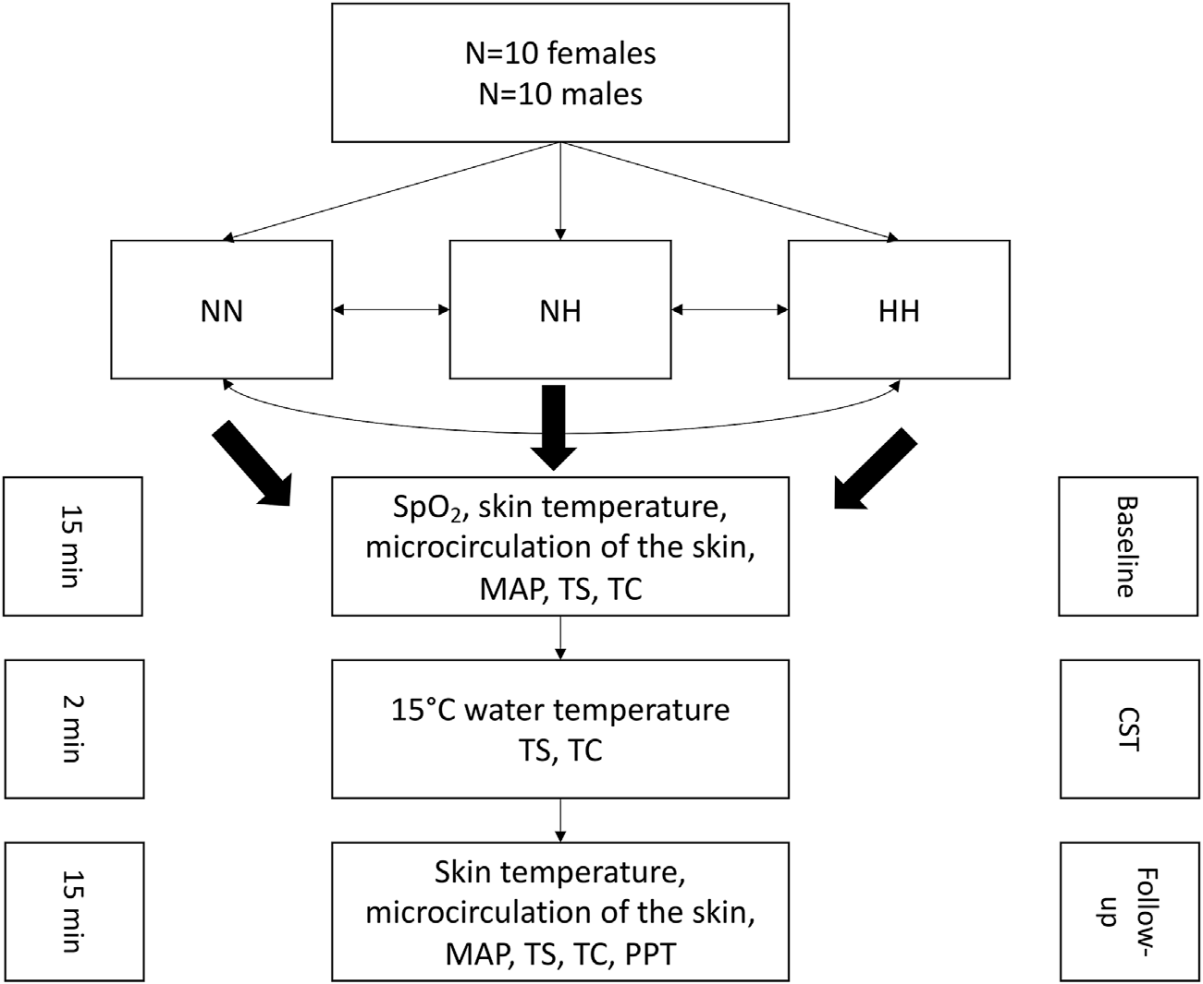
Schematic representation of the experimental protocol. NN: normobaric normoxia, NH: normobaric hypoxia, HH: hypobaric hypoxia, MAP: mean arterial pressure, TS: thermal sensation, TC: thermal comfort, CST: cold-stress test, PPT: pressure pain threshold.

After completion of the baseline measurements, the participant’s right hand was immersed in cold water for 2 min for the cold-stress test (CST), where SpO_2_, skin temperature, thermal sensation and comfort were assessed. Directly after the CST, the follow-up measurements were conducted which comprised SpO_2_, the hands’ skin temperature, microcirculation, blood pressure, thermal sensation- and comfort and the pressure pain threshold (PPT). The follow-up measurements were conducted in 5 min intervals up to 15 min post-cooling. The total duration for each measurement in each condition was around 45 min. A washout period of at least 1 week was between the conditions. A schematic representation of the test protocol is presented in Figure 1.

### 2.4 Cold-stress test

The environmental temperature in the laboratory and the mountain hut was intended to be neutral for the participants to avoid heat conservation (resulting in vasoconstriction of the hands’ skin) and heat loss (resulting in vasodilation of the hand’s skin). As thermoneutrality varies among individuals (Boudewyns, 1985), participants were allowed to adjust their clothing layers to achieve their thermal comfort at baseline. Before the beginning of the CST, the participant’s right hand was placed into a thin small plastic to avoid direct water contact with the skin. Then, the right hand was immersed up to the wrist for 2 min at a water temperature of 15°C in a seated position. The water temperature was monitored with a thermometer (Voltcraft, MT52, Wollerau, Switzerland). The participants were not allowed to actively move their hand during the CST but to remain as still as possible. This CST protocol has been demonstrated to be reliable to assess cold sensitization in healthy participants (House et al., 2015).

### 2.5 Peripheral oxygen saturation

Oxygen saturation in the blood was measured using pulse oximetry (Nonin 7500, Nonin medical b.v., Amsterdam, Netherlands) with an accuracy of ± 2%, from the left index finger. Pulse oximetry is a non-invasive method that passes red and infrared light through perfused tissue and detects the fluctuating signals caused by arterial pulses. Well-oxygenated blood is bright red, while poorly oxygenated blood is dark red. The pulse oximeter determines functional oxygen saturation of arterial hemoglobin (SpO_2_) from this colour difference by measuring the ratio of absorbed red and infrared light as volume fluctuates with each pulse. SpO_2_ measurement was conducted after the resting period until a stable value was reached. Pulse oximeters provide an estimate of arterial haemoglobin oxygen saturation, that is, the percentage of haemoglobin binding sites that are occupied at any one time by oxygen (Luks and Swenson, 2011). Deoxygenated and oxygenated haemoglobin absorb light at different wavelengths (660 and 940 nm respectively), and the absorbed light is processed by a proprietary algorithm in the pulse oximeter to display a saturation value (Torp et al., 2022). SpO_2_ was measured throughout each experimental trial and the mean value was taken to assess the difference between sexes in the different environments. Pulse oximetry has been demonstrated to be a valid measurement tool until a desaturation value of 85% is reached in a hypoxic environment (Kolb et al., 2004).

### 2.6 Local skin temperature

The skin temperature of the right dorsal hand was assessed using a conductive iButton (model: DS1922L) system (iButton, Maxim Integrated). The temperature logger was taped on the skin, centred over the third metacarpal bone. To avoid water contact, the participant’s hand was placed into a thin plastic bag during the CST. Skin temperature was assessed at baseline, during the CST in 1 min intervals (up to 2 min), and after the CST in 5 min intervals (point measurements). The change in local skin temperature (∆skin temperature [°C]) from baseline was then calculated and used for the analysis. It has been demonstrated that the iButton system is a valid and reliable instrument for measuring skin temperature in humans (Hasselberg et al., 2013).

### 2.7 Microcirculation of the skin

The microcirculation of the skin of the right dorsal hand was assessed with a calibrated laser speckle contrast imaging device (moorFLPI2, Moor instruments, Millwey, United Kingdom). The dorsal part of the hand was used because the skin temperature of the dorsal and palmar part of the hand have been shown to strongly correlate. To obtain standardized and valid perfusion values, the micro-vascularization of the right dorsal hand was investigated up to the wrist. Due to good temporal and spatial resolutions with a high frame rate, laser speckle contrast imaging allows measurements of acute changes in superficial skin blood flow over wide skin areas with very good inter-day reproducibility compared to traditional assessment technologies such as laser Doppler perfusion imaging and laser Doppler flowmetry (Roustit et al., 2010; Cracowski and Roustit, 2016). Cutaneous vascular conductance (CVC) was calculated at baseline, and after the CST in 5 min intervals (point measurement) from normalized microcirculation (flux) and MAP (mmHg) values. The normalized change in CVC (∆CVC [flux^.^MAP^−1^, flux^.^mmHg^−1^]) from baseline was then used for the analysis. A representative picture series of *n* = 1 female and *n* = 1 male participant of the microcirculation assessment in one condition (normobaric normoxia) can be seen in the Supplementary Figure S1.

### 2.8 Blood pressure measurement

Blood pressure was measured from the left brachial artery using an automated sphygmomanometer monitor (Beurer BM77, Beurer GmbH, Ulm, Germany). Mean arterial pressure (MAP, in mmHg) was calculated (Crisafulli et al., 2003) and assessed at baseline and after the CST in 5 min intervals (point measurements). Then, the change in MAP (∆MAP) from baseline was used for the analysis.

### 2.9 Thermal sensation and thermal comfort

The ratings of thermal sensation and thermal comfort were conducted according to ISO 10551 standards (International Organization for Standardization, 1995). The participants had to rate their thermal sensation according to the following scale: 4 = very hot, 3 = hot, 2 = warm, 1 = slightly warm, 0 = neutral, −1 = slightly cool, −2 = cool, −3 = cold, −4 = very cold. The scale to rate the individual thermal comfort consisted of: 0 = comfortable, 1 = slightly uncomfortable, 2 = uncomfortable, 3 = very uncomfortable, 4 = extremely uncomfortable. Thermal sensation and comfort were assessed at baseline, during the CST in 1 min intervals (up to 2 min), and after the CST in 5 min intervals (up to 15 min).

### 2.10 Pressure pain threshold

The PPT was determined using a handheld algometer with a probe area of 0.8 cm^2^. The pressure increased linearly, by around 20 kPa/sec. The measurements were performed at 1 cm distal from the medial knee joint line with the knee flexed at 90°. The participants were not allowed to see the algometer display at any moment and, as soon as the participants experienced a painful sensation, they said “stop”. The PPT assessment was always conducted by the same researcher. The first measurement in each environmental condition was performed at baseline and was considered only as a trial. The PPT measurement at the end of the CST follow-up time point (after 15 min) was used for the analysis. Pressure algometry has been demonstrated to have excellent reliability when applied to the medial part of the knee (Pelfort et al., 2015).

### 2.11 Statistical analysis

The assumption of normality was assessed using the Shapiro-Wilk test. A one-way ANOVA was performed for each environment (NN, NH, HH) to assess the differences between sexes (females, males) for SpO_2_, PPT, ∆CVC, and ∆skin temperature (mean ± SD). The Kruskal-Wallis ANOVA was used to analyse the differences between the sexes (females, males) for each environment (NN, NH, HH) across time for thermal sensation and thermal comfort (median±SE). The statistical analyses were performed using the Statistical Package for the Social Sciences (SPSS), version 28.0 (SPSS Inc., Armonk, United States) with the level of significance set at *p* < 0.05.

## 3 Results

### 3.1 Peripheral oxygen saturation

**FIGURE 2:**
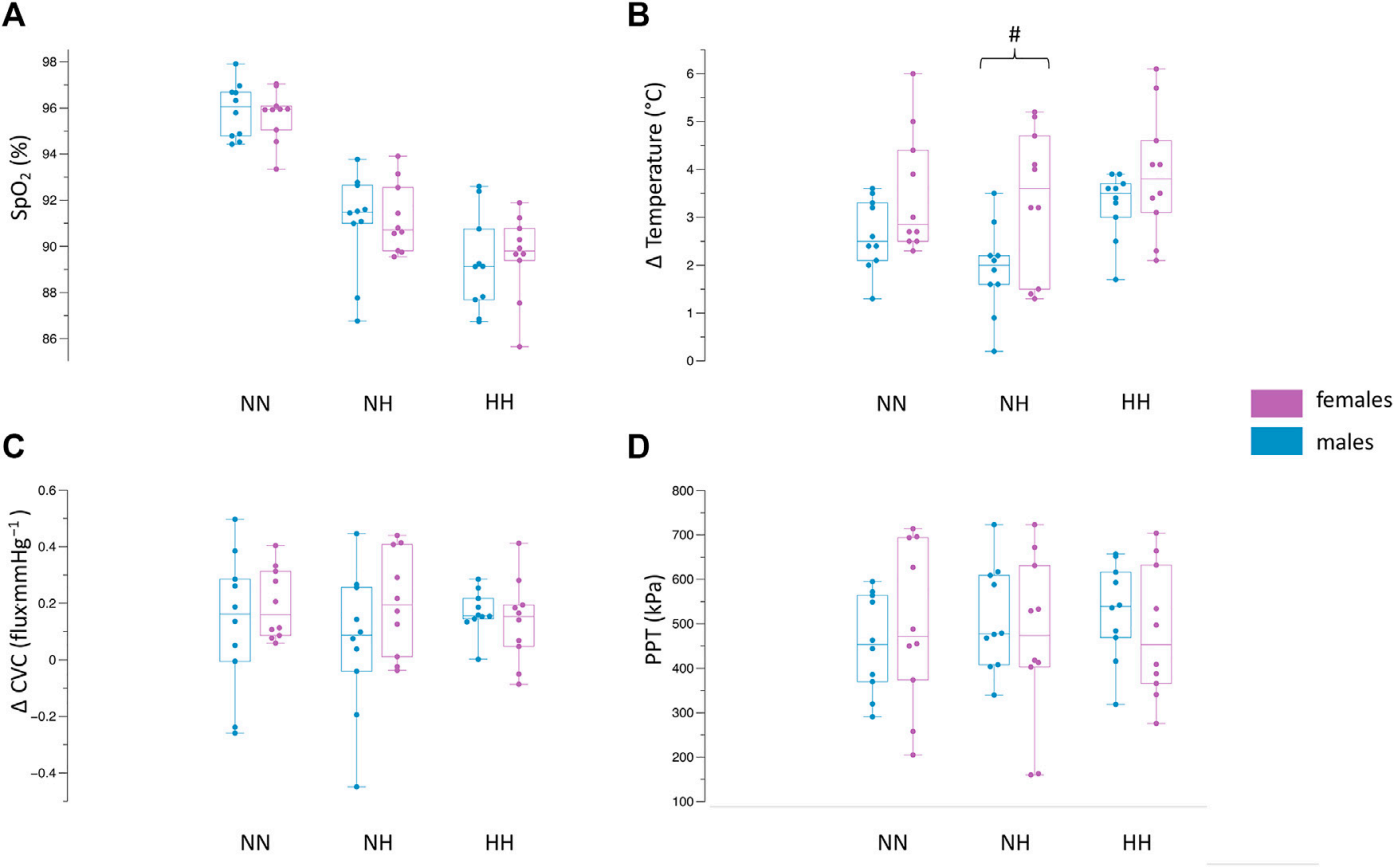
Differences between females and males in NN, NH and HH for SpO2 **(A)**, Δ skin temperature **(B)**, Δ CVC **(C)**, PPT **(D)**. Values demonstrate the median, iqr, min, max values. Dots indicate the individual values of each participant. #*p* = 0.01 between females and males.

For SpO_2_ (Figure 2A), no differences were observed between females and males in NN (females: 95.6 ± 1.1%, males: 95.8 ± 1.2%, *p* = 0.68), NH (females:91.2 ± 1.5%, males: 91.0 ± 2.1%, *p* = 0.83) or HH (females: 89.6 ± 1.8%, males: 89.2 ± 2.1%, *p* = 0.68).

### 3.2 Local skin temperature

The results of the change in skin temperature can be seen in Figure 2B. The skin temperature values at baseline, the end of the CST (2 min) and the end of the follow-up period (15 min) can be seen in Table 2. The drop in skin temperature due to the CST was higher (*p* = 0.01) during the NH environment in the female population compared to the males (females ∆skin temperature: 3.3 ± 1.5°C, males ∆skin temperature: 1.9 ± 0.9°C). In NN, no significant differences (*p* = 0.08) could be observed between females (∆skin temperature: 3.4 ± 2.2°C) and males (∆skin temperature: 2.6 ± 0.7°C). Also in the HH environment, there were no differences (*p* = 0.1) for the changes in skin temperature between females and males (∆skin temperature: 3.9 ± 1.3°C vs. 3.2 ± 0.6°C).

**TABLE 2:** Descriptive data in function of time.

	Baseline	CST 2 min	Follow-up 15 min
NN temperature (°C)			
Males	31.3 ± 0.4	25.7 ± 1.1	30.6 ± 1.5
Females	30.2 ± 2.2	24.7 ± 1.7	27.5 ± 1.4
NH temperature (°C)			
Males	30.4 ± 0.8	25.6 ± 1.1	30.2 ± 1.9
Females	30.3 ± 2.8	25.0 ± 1.7	27.5 ± 1.3
HH temperature (°C)			
Males	29.7 ± 1.7	23.6 ± 1.0	27.7 ± 2.9
Females	29.3 ± 1.4	23.6 ± 0.7	25.9 ± 1.1
NN microcirculation (flux)			
Males	73.8 ± 27.6	71.0 ± 16.9	59.0 ± 19.2
Females	50.3 ± 27.3	50.1 ± 23.2	36.0 ± 23.4
NH microcirculation (flux)			
Males	71.4 ± 25.2	71.7 ± 13.6	67.4 ± 37.3
Females	52.3 ± 27.0	49.0 ± 18.4	32.4 ± 14.4
HH microcirculation (flux)			
Males	98.1 ± 70.2	96.1 ± 35.2	77.7 ± 65.7
Females	44.3 ± 19.1	53.6 ± 21.1	29.5 ± 13.3
NN MAP (mmHg)			
Males	94.2 ± 6.6	95.5 ± 6.1	94.9 ± 4.4
Females	94.1 ± 9.7	96.8 ± 9.9	95.6 ± 12.1
NH MAP (mmHg)			
Males	93.9 ± 5.4	95.1 ± 5.9	93.5 ± 5.1
Females	93.6 ± 10.7	95.5 ± 10.9	96.5 ± 10.7
HH MAP (mmHg)			
Males	98.1 ± 8.1	99.7 ± 9.4	95.7 ± 7.0
Females	97.5 ± 9.4	98.3 ± 8.7	96.3 ± 9.2
NN TS (4 to −4)			
Males	1.0 ± 0.1	−2.5 ± 0.3	0.0 ± 0.1
Females	0.0 ± 0.3	−2.5 ± 0.2	0.0 ± 0.2
NH TS (4 to −4)			
Males	1.0 ± 0.1	−2.5 ± 0.2	0.0 ± 0.2
Females	0.5 ± 0.3	−3.0 ± 0.3	0.0 ± 0.2
HH TS (4 to −4)			
Males	−1.0 ± 0.2	−3.0 ± 0.2	−1.0 ± 0.2
Females	−1.0 ± 0.4	−3.0 ± 0.4	−1.0 ± 0.3
NN TC (0–4)			
Males	0.0 ± 0.1	1.0 ± 0.1	0.0 ± 0.0
Females	0.0 ± 0.2	1.0 ± 0.2	0.0 ± 0.1
NH TC (0–4)			
Males	0.0 ± 0.0	1.0 ± 0.1	0.0 ± 0.0
Females	0.0 ± 0.2	1.0 ± 0.2	0.0 ± 0.1
HH TC (0–4)			
Males	0.0 ± 0.2	2.0 ± 0.2	0.0 ± 0.1
Females	0.5 ± 0.2	1.0 ± 0.2	0.0 ± 0.1

Abbreviations: CST, cold-stress test; NN, normobaric normoxia; NH, normobaric hypoxia; HH, hypobaric hypoxia; MAP, mean arterial pressure; TS, thermal sensation; TC, thermal comfort, values are means ± SD (temperature, microcirculation, MAP) and medians±SE (TS, TC).

### 3.3 Cutaneous vascular conductance

The analysis (Figure 2C) revealed no differences between females and males for ∆CVC in NN (0.19 ± 0.12 flux^.^mmHg^−1^ vs. 0.13 ± 0.24 flux^.^mmHg^−1^, *p* = 0.45), NH (0.20 ± 0.18 flux^.^mmHg^−1^ vs. 0.06 ± 0.25 flux^.^mmHg^−1^, *p* = 0.18) and HH (0.13 ± 0.14 flux^.^mmHg^−1^ vs. 0.16 ± 0.07 flux^.^mmHg^−1^, *p* = 0.54). The absolute values for MAP and microcirculation can be seen in Table 2.

### 3.4 Pain pressure threshold

The mean pain pressure threshold (Figure 2D) was not different between females and males in NN (496.1 ± 183.7 kPa vs. 455.4 ± 111.2 kPa, *p* = 0.55), NH (464.5 ± 193.9 kPa vs. 511.2 ± 118.8 kPa, *p* = 0.52) and HH (481.1 ± 148.2 kPa vs. 528.4 ± 108.4 kPa, *p* = 0.55).

### 3.5 Thermal sensation and thermal comfort

**FIGURE 3:**
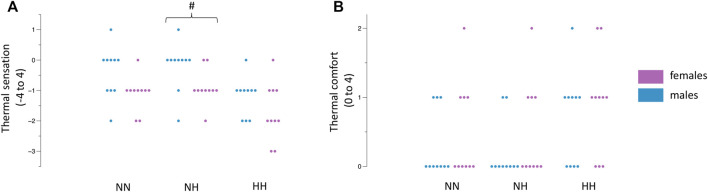
Differences between females and males in NN, NH and HH for thermal sensation **(A)** and thermal comfort **(B)**. Dots indicate the individual values of each participant. #*p* = 0.02 between females and males.

The female participants rated their immersed hand to feel colder during NH (−0.9 ± 0.5 vs. -0.2 ± 0.7, *p* = 0.02, Figure 3A) whilst no differences were observed in NN (−1.1 ± 5.6 vs. −0.4 ± 0.8, *p* = 0.05) and HH (−1.7 ± 0.9 vs. −1.2 ± 0.6, *p* = 0.18). Thermal comfort ratings were not different between females and males (all p >0.05, Figure 3B). The values across time can be observed in Table 2.

## 4 Discussion

This study aimed to investigate potential sex differences in the physiological and perceptual response to a CST in a NN, NH and HH environment. The main findings of this study are, that the skin’s temperature drop (Figure 2B) and the perception of cold (Figure 3A), in response to the CST, were more pronounced in females compared to the male population only in the NH environment. There were no sex differences present in SpO_2_, CVC, PPT, and thermal comfort in response to the CST in NN, NH and the HH environment.

The current findings reveal that the CST induce no differences between sexes under normoxic conditions. This finding is partially in line with another study that investigated sex differences during a CST (Kilgour and Carvalho, 1994). In this study, the investigators conducted a more severe CST of the hand (5–7°C for 6 min) and concluded that the skin’s finger temperature didn’t differ between females (∆females: 16.8 ± 1.1°C, ∆males: 18.4 ± 0.9°C). However, the rise in systolic and diastolic pressure was maintained for a longer time in males compared to females in this study, which was attributed to the changes in systemic vascular resistance and the negligible alteration in cardiac output (Kilgour and Carvalho, 1994). In contrast, another research group found significant lower skin temperature values in women (25.2 ± 1.6°C) compared to men (27.1 ± 1.4°C) after a hand CST (15°C for 15 min) (Bartelink et al., 1993). Generally, the response to acute cooling is peripheral vasoconstriction, which is a necessary, physiological response to prevent heat loss, but results in a strong temperature decrease in the skin (Daanen, 2003). However, findings from the late 1970s have indicated that females have a severe thermoregulatory disadvantage compared to males in cold environments due to their increased heat loss-to-production (surface-to-mass) ratio (Burse, 1979). Although women have in general a higher percentage of body fat than men (Blaak, 2001), this insulative effect does not provide an advantage in preventing heat loss from the extremities. The less pronounced and less prolonged cold-induced vasoconstriction in males and the changes in female hormonal levels might contribute to the different responses between sexes (Bartelink et al., 1993). A plausible explanation is the influence of the female hormones on the peripheral adrenergic synapses. An elevation of estrogen levels have been associated with an upregulation of vasoconstrictive α-adrenoreceptors (Bartelink et al., 1993). It is also described that the females’ basal hand blood flow is lower compared to men (Cooke et al., 1990). These authors concluded that the increase in sympathetic activation is the main factor for sex differences regarding blood flow differences, rather than local structural differences (Cooke et al., 1990). In summary, previous research has shown that variations during cooling are related to the above-mentioned sex differences and morphological differences in the extremities (Havenith et al., 1992; Jay and Havenith, 2004; Lunt and Tipton, 2014).

Interestingly, our results show a higher skin temperature drop in the hands in females compared to males, only in normobaric hypoxia but not in hypobaric hypoxia. The decrease in arterial PO_2_ stimulates peripheral and central chemoreceptors. This stimulation occurs when the inspired PO_2_ is lowered to approximately 122 mmHg or at an altitude of 1,524 m (Institute of Medicine Committee on Military Nutrition, 1996), which also occurred in our study at an altitude of around 3,000 m. One of the several physiological responses that occur under acute hypoxia is the stimulation of the sympathetic nervous system, a principal secretion of catecholamines like norepinephrine (Brooks et al., 1991; Mazzeo et al., 1991). By stimulating the chemoreceptors, which are responsible for the sympathetic drive, the resulting vasoconstrictive effect is responsible to maintain blood flow (e.g., coronary and cerebral) to the tissues (Heistad and Abboud, 1980; Hansen and Sander, 2003). Interestingly, one study result showed that the latency for achieving peak response to isocapnic hypoxaemia was significantly shorter in females compare with men (Jones et al., 1999). Consistent with the more rapid responses observed in women, females show increased dopamine and NA turnover in carotid body and brainstem NA cell groups in response to hypoxia compared to males, however these results are based on animal models (Pequignot et al., 1997). It is well established that a hypoxic environment triggers changes in autonomic nervous system reactions (Hainsworth et al., 2007). In this context, interesting results were observed from a research group, investigating sex differences during hypoxia on autonomic nervous system responses. The results indicated that males tend to have higher sympathetic responses compared to females (Botek et al., 2018), which is in accordance to our results. In line with the literature (Millet et al., 2012), our results suggest that the HH environment seems to be a more severe environment compared to NH, and triggered the sympathetic nervous system more in males compared to females (Botek et al., 2018). Our results show a higher skin temperature drop in HH compared to NH in males (males HH: ∆3.2 ± 0.6°C vs. males NH: ∆1.9 ± 0.9°C) compared to the (females HH: ∆3.9 ± 1.3°C vs. females NH: ∆3.3 ± 1.5°C). From another perspective, the more pronounced skin temperature drop in NH compared to HH in the female group might be due to the instant hypoxic stimulus. The hypoxic stimulus occurred only instantly in the NH set-up, once the participants breathed the hypoxic air through the mask system. In the HH group, the hypoxic stimulus occurred in a more moderate way but over a longer time period, because travelling to the terrestrial target altitude took much more time (Figure 1). Therefore, it can’t be excluded that the instant hypoxic effect might have influenced the current results. It is also conceivable that the additional stimulation of the adrenergic system by the acute hypoxia intensifies the cold-induced vasoconstriction and vice versa. Indeed, results from earlier studies also show that systemic hypoxaemia leads, compared to normoxic conditions, to a blunted cold-induced vasodilation and skin temperature (Takeoka et al., 1993; Daanen and van Ruiten, 2000). The females rated the water temperature to feel colder compared to the men in the NH group. This finding is in line with the findings of a research group where females and males underwent a thermal stress test (Averbeck et al., 2017).In this study, females were more sensitive to thermal detection and also thermal pain threshold. Interestingly, independent of sex, thermal detection thresholds were dependent on the baseline temperature, demonstrating complex processing of “cold” and “warm” inputs in thermal perception (Averbeck et al., 2017).

A limitation of this study is, that the participants’ core temperature was not assessed. Other researchers have demonstrated, that a slight increase in core temperature leads to an enhanced cold-induced vasodilation (Daanen et al., 1997). Another limitation is, that the hypoxic dose was different between NH and HH. Whilst the participants in NH were exposed to the targeted hypoxic level instantly, in HH the systemic hypoxaemia occurred over a longer time until the equivalent hypoxic level was reached. It is possible that the age factor might attributed to the current results, as the females were statistically younger (*p* = 0.047) compared to the males. Age has been demonstrated to influence the sympathetic tone in young women to physiological stressors (Miller et al., 2019). Albeit our aim was to have the exact same P_i_O_2_ in NH and HH, the hypoxic dose was slightly higher in NH compared to HH (around 5 mmHg), which should be taken into account. Future studies should consider increasing the normobaric hypoxic stimulus in a time period comparable to that of summit ascent under real conditions. Furthermore, future studies should consider investigating sex differences between normobaric and hypobaric conditions under more severe hypoxic environments.

## 5 Conclusion

The cold-stress test led to sex differences only under normobaric hypoxia, where the females’ skin temperature dropped to a larger amount, and the thermal sensation of cold was higher, compared to the males. Females and males showed similar physiological and perceptual responses to the cold-stress test in normobaric normoxia, normobaric- and hypobaric hypoxia. These findings might help to better understand the physiological and perceptual responses to hypoxic and cold stressors between females and males.

## Data Availability

The raw data supporting the conclusions of this article will be made available by the authors, without undue reservation.
