# Supplementary figures and images for: Sex differences during a cold-stress test in normobaric and hypobaric hypoxia: A randomized controlled crossover study
Source: Front Physiol. 2022 Sep 23;13:998665. doi: 10.3389/fphys.2022.998665 (PMC9549379; doi:10.3389/fphys.2022.998665)

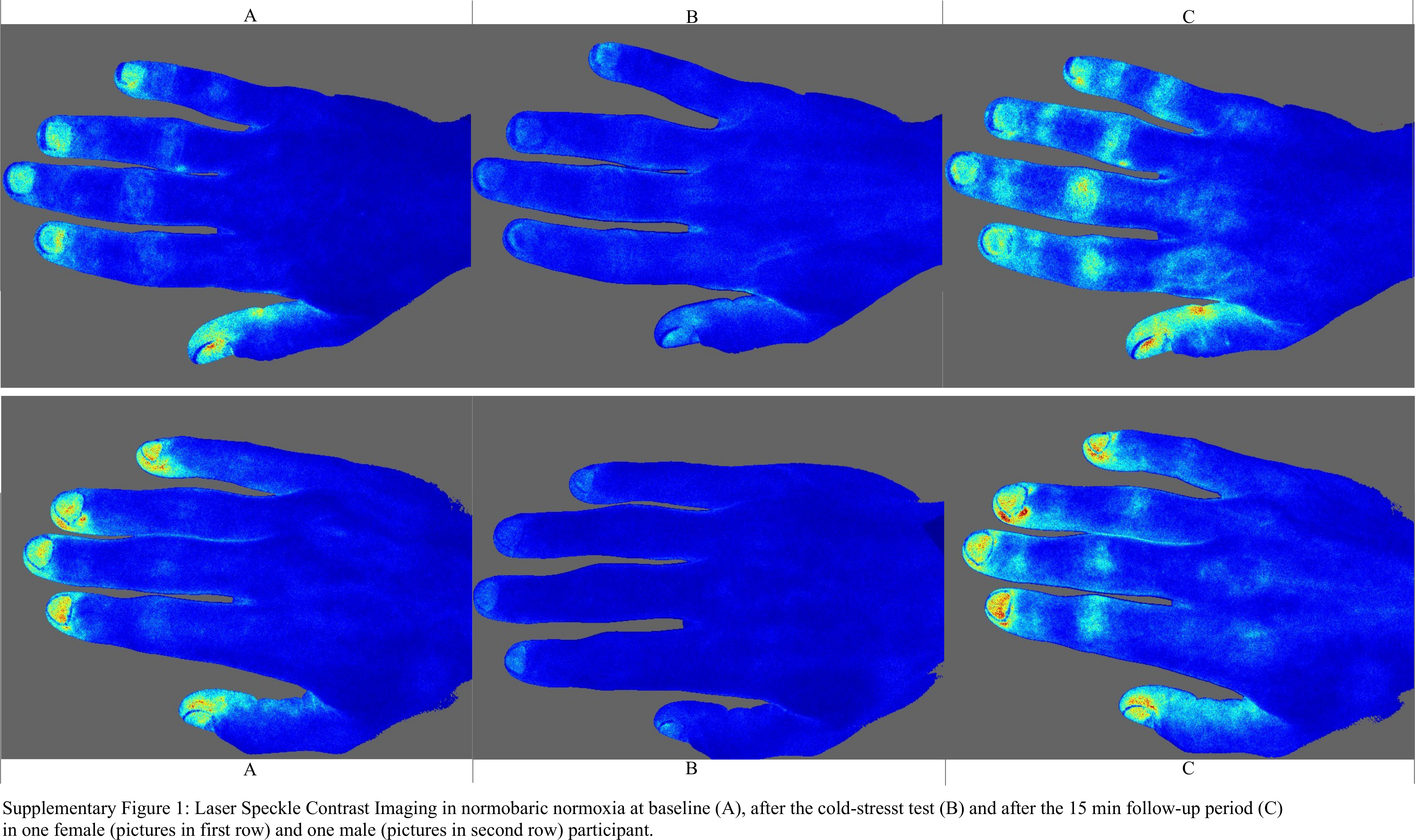

Supplement: Supplementary file 1 [file Image1.TIFF]
